# Stimulation-induced differential redistributions of clathrin and clathrin-coated vesicles in axons compared to soma/dendrites

**DOI:** 10.1186/s13041-020-00683-5

**Published:** 2020-10-16

**Authors:** Jung-Hwa Tao-Cheng

**Affiliations:** NINDS Electron Microscopy Facility, National Institute of Neurological Disorders and Stroke, National Institutes of Health, Bethesda, MD 20892 USA

**Keywords:** Electron microscopy, Clathrin-coated vesicles, Clathrin-coated pits, Endocytosis, Synaptic vesicle, Multivesicular body, Glutamate receptors, Transferrin receptors

## Abstract

Clathrin-mediated endocytosis plays an important role in the recycling of synaptic vesicle in presynaptic terminals, and in the recycling of transmitter receptors in neuronal soma/dendrites. The present study uses electron microscopy (EM) and immunogold EM to document the different categories of clathrin-coated vesicles (CCV) and pits (CCP) in axons compared to soma/dendrites, and the depolarization-induced redistribution of clathrin in these two polarized compartments of the neuron. The size of CCVs in presynaptic terminals (~ 40 nm; similar to the size of synaptic vesicles) is considerably smaller than the size of CCVs in soma/dendrites (~ 90 nm). Furthermore, neuronal stimulation induces an increase in the number of CCV/CCP in presynaptic terminals, but a decrease in soma/dendrites. Immunogold labeling of clathrin revealed that in presynaptic terminals under resting conditions, the majority of clathrin molecules are unassembled and concentrated outside of synaptic vesicle clusters. Upon depolarization with high K^+^, label for clathrin became scattered among de-clustered synaptic vesicles and moved closer to the presynaptic active zone. In contrast to axons, clathrin-labeled CCVs and CCPs were prominent in soma/dendrites under resting conditions, and became inconspicuous upon depolarization with high K^+^. Thus, EM examination suggests that the regulation and mechanism of clathrin-mediated endocytosis differ between axon and dendrite, and that clathrin redistributes differently in these two neuronal compartments upon depolarization.

## Introduction

Clathrin-mediated endocytosis (CME) is a fundamental process of all mammalian cells that enables internalization of receptors and cargos from the plasma membrane (PM) [1, 2]. Clathrin molecules exist as individual triskelia in the cytoplasm, and are assembled on PM via adaptor and accessary proteins forming a clathrin-coated pit (CCP), which can then be pinched off to become a clathrin-coated vesicle (CCV) in the cytoplasm [1, 2]. Clathrin eventually sheds from CCV and becomes disassembled in the cytoplasm.

In neurons, CME plays an important role in synaptic vesicle (SV) recycling in axon terminals [3, 4], preventing unlimited enlargement of surface membrane area due to exocytosis of SVs during stimulation. On the other hand, in soma/dendrites of neurons, CME is involved in the internalization of transmitter receptors, as well as other receptors and cargos [5, 6]. Notably, CCVs in the brain are smaller in axons than in dendrites, with a size difference at about twofold [7]. Although the size of axonal CCV in presynaptic terminals has been extensively studied [4], few studies have measured the size of dendritic CCV. Here, the size of somal/dendritic CCVs was measured from perfusion-fixed mouse brains as well as from 4 day to 3 week-old rat dissociated hippocampal neuronal cultures to further document this size difference in axon vs. dendrites at various developmental stages.

The present study also investigated the potential ultrastructural identity of stable “hot spots” of endocytic sites [5, 6] on soma/dendrites, whether the formation of somal/dendritic CCPs is influenced by the juxtaposed cellular elements, and compared the structural organization of the clathrin-labeled patches on multivesicular body (MVB) to those of CCVs.

Activity-induced increase in the formation of CCVs in presynaptic terminals has been reported in different experimental systems [3, 8, 9]. The present study further examined the effect of a delay in perfusion fixation on rodent brains to see if such an ischemia-like stimulation affects the formation of CCVs in presynaptic terminals. Additionally, structural changes in CCVs and CCPs of 3 wk-old dissociated cultures were compared under control and depolarizing conditions, with particular attention to difference in response between axons and dendrites.

Previous immunogold EM studies have demonstrated that unassembled clathrin molecule itself is not visible until many of them assemble to form a coat on CCPs and CCVs [7, 10]. While EM can capture only one static image at a time, light microscopy [LM] studies can trace green fluorescent protein (GFP)-tagged clathrin [5] or pH-sensitive super-ecliptic pHluorin (SEP)-tagged receptors [6] live, capturing the formation and fission of CCP and CCV which contain concentrated labeling. However, most LM study on clathrin in neurons have focused on soma/dendrites, perhaps because the larger CCVs in soma/dendrites made them easier to image than the smaller sized CCVs in axon terminals. The present study used immunogold EM to examine the distribution of endogenous clathrin, both as unassembled molecules in the cytoplasm and as assembled clathrin coat on CCVs and CCPs in 3 week-old dissociated hippocampal neuronal cultures. Redistribution of clathrin molecules upon depolarization with high K^+^ was quantified for both the axon terminals and the soma/dendrites to illustrate the different responses in these two compartments.

## Methods

### Antibodies

Mouse monoclonal antibody (mouse mAb) against clathrin (clone X22, 1:200–500) and AP2 (a clathrin adaptor protein-2, clone AP6, 1:100–200) were from Affinity Bioreagents (Golden, CO, USA); mouse mAb against transferrin receptor (TfR, clone OX-26) was from Chemicon (Temecula, CA, USA). Controls for specificity of immunolabeling include omitting the primary antibody and using the different primary antibodies as controls for each other.

### Preparation, treatment, fixation and pre-embedding immunogold labeling of rat dissociated hippocampal neuronal cultures

Most samples were from previously published reports [11–14] and reexamined here for structural changes of CCV and CCP, and for distribution of clathrin under different conditions. Briefly, cell cultures were prepared from embryonic 20-day-old rat fetuses by papain dissociation, and then plated with or without a glial feeder cultures, and examined at 3–6 or 19–28 days in vitro (DIV). Depolarization-related experiments were carried out with ~ 3 week-old cultures.

Culture dishes were placed on a floating platform in a water bath maintained at 37˚C for all experiments. Control incubation medium was HEPES-based Krebs Ringer at pH 7.4. High K^+^ medium was at 90 mM KCl, with osmolarity compensated by reducing the concentration of NaCl. Cell cultures were washed with control medium and treated for 2–3 min with either control or high K^+^ media and then fixed immediately.

For optimal structural preservation, cells were fixed with 4% glutaraldehyde in 0.1 M cacodylate buffer at pH 7.4 for 30 min to 1 h at room temperature and then stored at 4˚C. Some samples were fixed with 4% glutaraldehyde plus 1% tannic acid in buffer. For pre-embedding immunogold labeling, cells were fixed with one of the following procedures: (1) 4% paraformaldehyde in phosphate buffered saline (PBS) for 30–60 min, (2) 4% paraformaldehyde and 0.02–0.1% glutaraldehyde for 30–45 min, (3) 1–2% acrolein in PBS for 1 min followed by 4% paraformaldehyde in PBS for 30–60 min.

Samples fixed for pre-embedding immunogold labeling were washed and permeabilized/blocked with 0.1% saponin/5% normal goat serum in PBS for 1 h, incubated with primary antibody for 1–2 h, incubated with secondary antibody conjugated to 1.4 nm gold particles (1:250, Nanogold from Nanoprobes, Yaphank, NY, USA) for 1 h, washed in water and silver enhanced (HQ silver enhancement kit, Nanoprobes) to make the small gold particles visible. All steps were carried out at room temperature.

### Perfusion fixation of rat and mouse brains

Most samples were from previously published reports [15, 16] and reexamined here for additional structural changes of CCV and CCP. Briefly, adult rats were deeply anesthetized with Nembutal, and mice from 1 to 3-month-old were deeply anesthetized with isoflurane. Animals were perfusion fixed through the heart with 2% glutaraldehyde +2% paraformaldehyde in 0.1 M sodium cacodylate buffer at pH 7.4, or first perfused with 3.75% acrolein +2% paraformaldehyde, then followed by 2% paraformaldehyde. The time interval starting from the moment the diaphragm was cut to the moment when the outflow from the atrium turned from blood to clear fixative was recorded. Those animals that were successfully perfused within 100 s were classified as “fast” perfusion. For the “delayed” perfusion experiments, phosphate-buffered saline containing calcium and magnesium was first perfused through the heart for 5–8 min before the start of the fixative. Neurons were under resting state after fast perfusion, and under ischemic excitatory conditions after delayed perfusion fixation [15, 16]. The perfusion-fixed brains were dissected and vibratomed into 100 µm thick coronal slices and stored in 2% glutaraldehyde in buffer at 4˚C.

### Electron microscopy

Most samples fixed with glutaraldehyde for structural analysis were post-fixed with 1% osmium tetroxide in 0.1 M cacodylate buffer for 1 h on ice, and stained with 1% uranyl acetate in acetate buffer at pH 5.0 overnight. Additionally, some samples were post fixed with “reduced osmium” (1% potassium ferricyanide + 1% osmium tetroxide) in 0.1 M cacodylate buffer for 1 h on ice.

Samples for immunogold labeling were treated with 0.2% osmium tetroxide in phosphate buffer for 30 min on ice, followed by 0.25% uranyl acetate in acetate buffer at pH 5.0 on ice for 30 min-1 h. Both types of samples (for structural study or for immunogold labeling) were dehydrated in a graded series of ethanol and embedded in epoxy resins. Thin sections were cut at ~ 70 nm and counterstained with uranyl acetate and lead citrate. Some blocks were serial sectioned and collected on single-slot grids. Images were photographed with a bottom-mounted digital CCD camera (AMT XR-100, Danvers, MA, USA).

### Morphometry

#### Identification of neuronal soma, dendrite, axon, and synapses

Identification of neuronal soma, dendrites, axons and synapses was based on criteria described in a classic EM atlas [17]. Neuronal somas can be unequivocally identified based on their structural characteristics [14], and primary dendrites can be traced from their connection to the soma. The present study focused on glutamatergic excitatory synapses, which are characterized by (1) clusters of SV in presynaptic axonal terminals, (2) the synaptic cleft with a uniform gap of 20 nm between the pre- and postsynaptic membranes, and (3) the postsynaptic density (PSD) on the dendritic element, facing the active zone of the presynaptic terminal [12]. Segments of axons and dendrites can, in turn, be unequivocally identified by tracing their connections from the synapses.

#### Measurements of size of clathrin-coated vesicles (CCV) in soma/dendrites of neurons

The diameter of a CCV was determined by measuring its maximal diameter (L1) and a second diameter (L2) taken from the midpoint of L1 perpendicular to L1 (Additional file 1a); its average diameter was defined as (L1 + L2)/2. Measurements were taken from the outside edge of the vesicle membrane, not including the clathrin coat.

#### Scoring of clathrin-coated pits (CCP) on plasma membrane of neuronal somas

The plasma membrane (PM) of each encountered neuronal soma was examined for presence of CCP (arrows in Additional file 1b), and the number of pits per soma was scored. At least 10 somas were scored for each sample to calculate an average value of number of CCP per soma.

#### Scoring of CCP and CCV within presynaptic terminals

CCPs and CCVs in presynaptic terminals were identified by the structural characteristics of assembled clathrin coat. Since the size of CCVs in presynaptic terminals is ~ 40 nm [4], each CCVs is typically contained within a single thin section which is ~ 70 nm in thickness. Every presynaptic terminal profile from archived images [12, 15, 16] was examined to score the number of CCVs and CCPs. The total number of CCPs and CCVs was pooled from every presynaptic terminals encountered, and a value per 100 presynaptic terminals was calculated for each sample.

#### Measurements of density and distance of label for clathrin in presynaptic terminals of dissociated hippocampal cultures

At least 4–5 openings of a 400-mesh, hexagonal grid with openings at ~ 50 µm were randomly selected from thin sections, and every synaptic profile of excitatory (asymmetric) synapses with a discernible cross section of the synaptic junction was photographed for measurements.

For labeling density, two perpendicular lines were drawn from the edge of the active zone (Additional file 2a) to a depth of 200 nm from the presynaptic membrane. This depth of 200 nm was arbitrarily set in step with the criteria of an earlier report on quantification of redistribution of presynaptic proteins near the active zone [12]. Area of measurement was then bordered by the presynaptic membrane and a dashed line drawn at the depth of 200 nm, parallel to the presynaptic membrane (Additional file 2a). All labels within this area were counted and divided by the length of this area, and expressed as number of labels/µm length of active zone. Distance of labels within this area was measured from the center of the black particles to the outer edge of the presynaptic membrane (arrows in Additional file 2b), and then plotted into histograms for each sample.

#### Quantitation of number of clathrin-labeled CCP and CCV near plasma membrane of neuronal soma and primary dendrites of dissociated hippocampal cultures

In samples labeled for clathrin, every encountered neuronal soma and its continuous primary dendrites was photographed. A “band” of cytoplasm 1 µm deep from the plasma membrane (PM) was marked as the measurement area (Additional file 3, section #1). Glogi complexes were excluded from measurement because Golgi-associated clathrin-coated vesicles are not involved in endocytosis at the PM.

Within the marked measurement area, every labeled CCP and CCV was counted and then the total was divided by the length of the band of cytoplasm, expressed as number of CCVs + CCPs / µm length of PM. The criteria for counting a cluster of label for clathrin as a CCV is that there are at least 5 particles of label within an area of 100 nm in diameter (circled in Additional file 3, sections #2 and 3).

#### Statistical analyses

Comparisons of number of CCVs and CCPs in presynaptic terminals and in neuronal soma/dendrite, and comparisons of mean density of label for clathrin at presynaptic terminals under different conditions were carried out with Student’s t-test. Comparisons of median distance of label for clathrin at presynaptic terminals were carried out with the non-parametric Wilcoxon test.

## Results

### CCVs are smaller in axons than in soma/dendrite

**Fig. 1:**
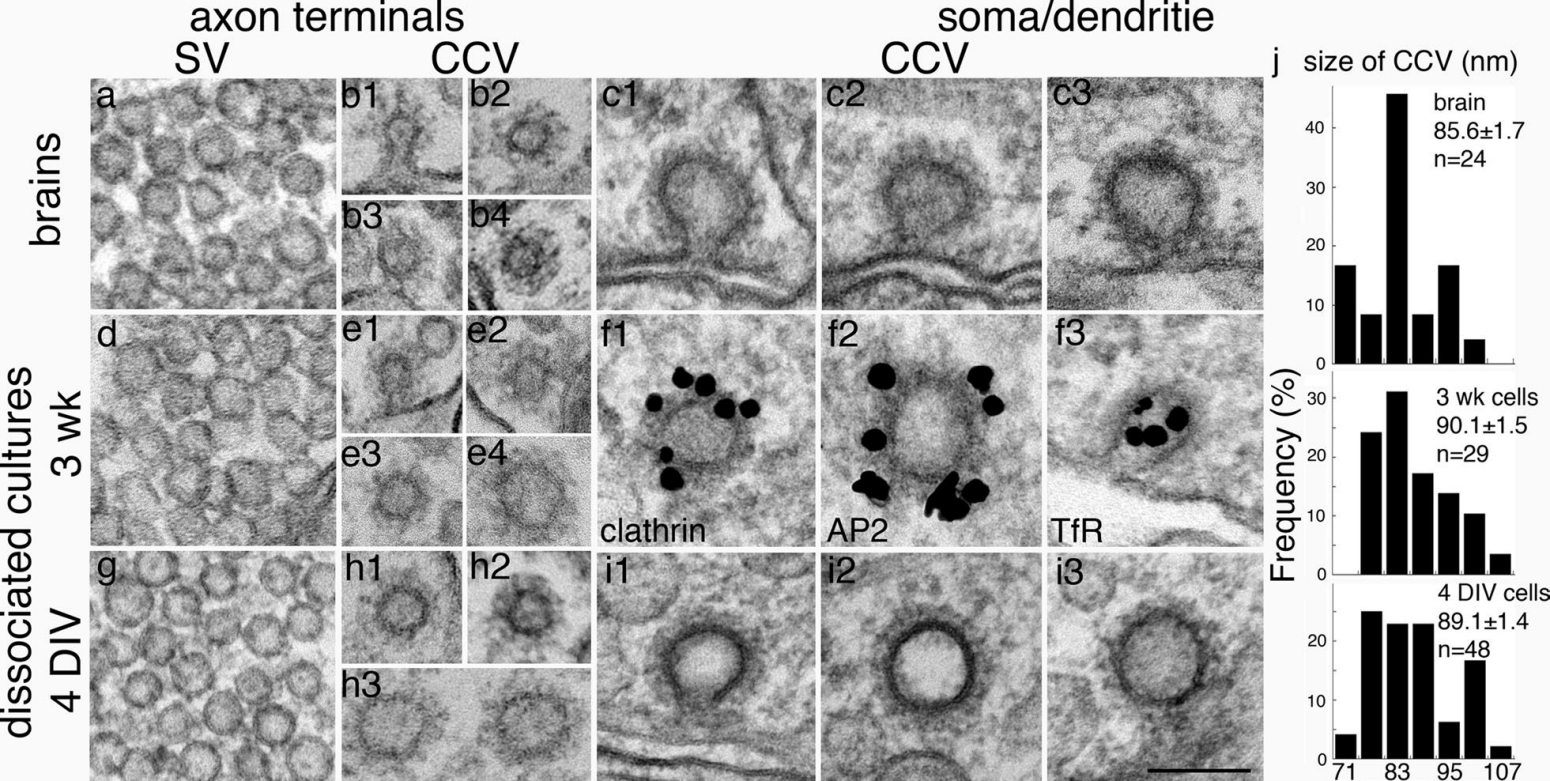
Clathrin-coated vesicles in axon and soma/dendrite are of different sizes. Images were sampled from perfusion-fixed mouse brains (top row: **a**–**c**), 3 wk-old (middle row: **d**–**f**) and 4 DIV (bottom row: **g**–**i**) dissociated rat hippocampal cultures. Synaptic vesicles (SV) were included as size references for CCV from the respective samples, and SVs from these three different groups of samples were of the same uniform size at ~ 40 nm in diameter (**a**, **d**, and **g**). CCVs in axon terminals from brains (**b1**–**b4**) were of the same size as SV. While the great majority of CCVs in axon terminals from dissociated cultures (**e1-3**; **h1-2**) were also of the same size as SV, some CCVs (**e4**; **h3**) were larger (~ 70 nm) than SV. In soma/dendrites (**c**, **f**, **i**), CCV were ~ 90 nm in diameter, much larger than those in the axon terminals. Immunogold labeling of 3 week-old cells illustrates that CCV in soma/dendrites labeled for clathrin (**f1**), AP2 (**f2**), and transferrin receptor (TfR, **f3**). **j** Histograms of size distribution of CCVs of soma/dendrites from brains (top panel), and dissociated neuronal cultures at 3 weeks (middle panel) and 4 days (bottom panel) in culture. The ranges of average diameter were similar (70–110 nm) among the three types of samples, and there was no statistical significance in mean values (ANOVA) or in median values (Wilcoxon test, median values at 85.8, 88.3, 88.3 nm, respectively). n = number of CCVs measured. Scale bar = 100 nm

One of the striking features of synaptic vesicles (SV) is the uniformity of their size [4, 18], and this uniformity is across species. Glutamatergic SVs in mammalian brains are all ~ 40 nm in diameter in mouse, rat, cat and monkey [17]. Examples of SVs from glutamatergic presynaptic terminals of different samples are shown in Fig. 1a, d, g. In perfusion-fixed 1–3 month-old mouse brains, the size of CCVs in presynaptic terminals (Fig. 1b1–4) is the same as that of SVs (Fig. 1a) at ~ 40 nm. The same is observed in perfusion-fixed adult rat brains (images not shown). This observation is as expected since SVs result from shedding of clathrin from CCVs [4, 18]. In dissociated rat cultures, the great majority of CCVs were also ~ 40 nm in diameter. However, there were occasional CCVs that were larger at ~ 70 nm (70.1 ± 1.0, n = 11), and they were more prevalent in younger cultures at 3–6 DIV (Fig. 1h3) than at 3 wk in culture (Fig. 1e4). This observation suggests that larger axonal CCV may be associated with developing axons.

Although the “thickness” of the clathrin coat was the same at ~ 15 nm for axonal and dendritic CCVs, the average diameter of CCVs in neuronal soma and dendrites was conspicuously larger (~ 90 nm; Fig. 1c, f, and i) than CCVs in presynaptic terminals. The size distributions of somal/dendritic CCVs from the three different experimental materials were plotted into histograms (Fig. 1j). Although the mean diameter of CCVs from perfusion-fixed mouse brains was somewhat smaller than those from dissociated cultures of rat neurons, these differences did not reach statistical significance.

As expected [1], immunogold labeling of dissociated cultures demonstrated that CCVs in both axons and dendrites specifically labeled for clathrin and AP2, clathrin adaptor protein-2. Examples of dendritic CCVs labeled for clathrin and AP2 are illustrated in Fig. 1f1 and f2, respectively, consistent with LM observations that these two proteins co-localize as concentrated puncta representing CCVs [5]. In contrast, label for transferrin receptor (TfR), a constitutively endocytosed membrane protein [1, 2], is present in dendritic CCVs (Fig. 1f3) but absent in axons [19], indicating differential sorting of cargos between axon and dendrite.

### Presence of clustered CCPs on somal/dendritic plasma membrane

**Fig. 2:**
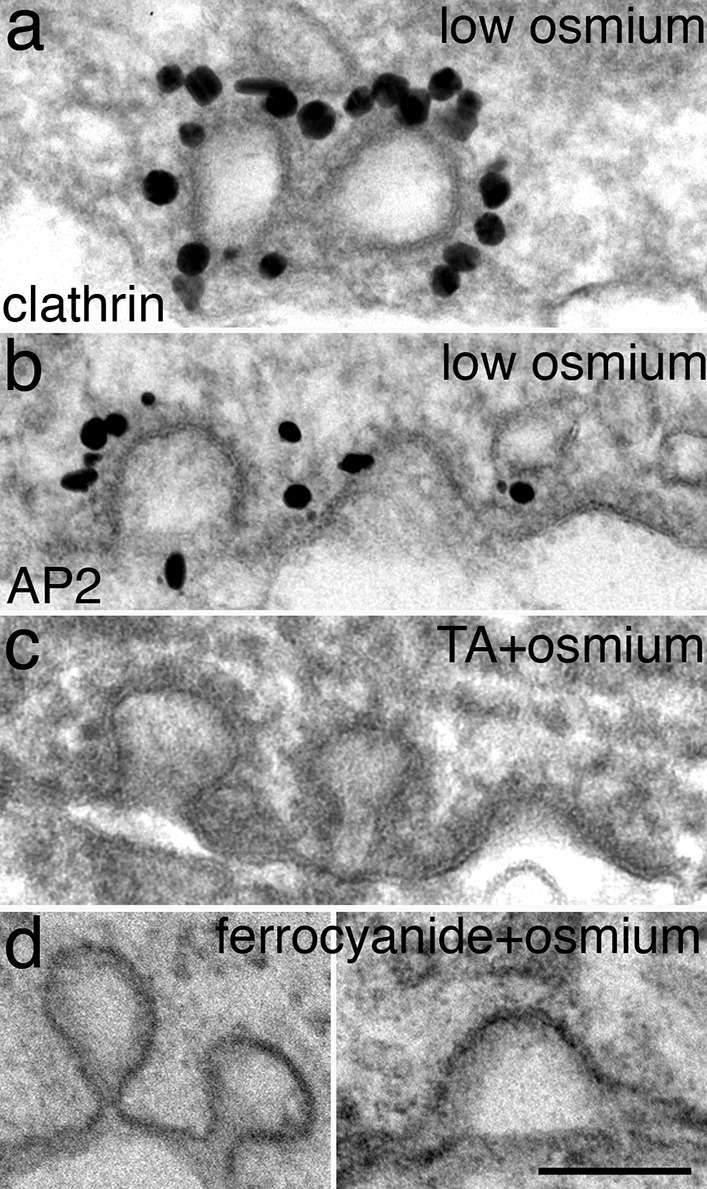
CCPs occasionally exist as multiples in close vicinity on somal/dendritic plasma membrane. The characteristic “coat” appearance was visible in samples treated with low concentrations of osmium tetroxide (0.2% in a, b), and labels for clathrin (**a**) and for AP2 (**b**) were specifically localized to the coat. The appearance of the coat was enhanced when tannic acid (1%) was added to fixative followed by regular osmium tetroxide (1% in **c**), and less conspicuous with “reduced osmium” treatment (1% potassium ferrocyanide + 1% osmium tetroxide in d). All samples were 3 week-old dissociated hippocampal cultures. Scale bar = 100 nm

Although the majority of CCPs in thin sections of neuronal soma/dendrites were captured as individual pits, clustered CCPs consisting of 2–3 pits within ~ 100 nm of each other were occasionally seen (Fig. 2). This finding is consistent with LM evidence suggesting that “hot spots” of GFP-tagged clathrin puncta support multiple endocytosis events [5], and that some optically stable endocytic sites can yield several CCVs within minutes [6].

The appearance of the clathrin coat was affected by specific EM fixation/staining reagents. Osmium tetroxide at a low concentration of 0.2% is enough to make the clathrin coat visible (Fig. 2a, b). Including tannic acid (1%) in the initial fixative along with glutaraldehyde enhanced the darkness of the clathrin coat (Fig. 2c), whereas adding 1% potassium ferrocyanide along with 1% osmium tetroxide in the postfixation reduced the visibility of the clathrin coat (Fig. 2d).

### Somal/dendritic CCPs can be juxtaposed to different cellular elements

**Fig. 3:**
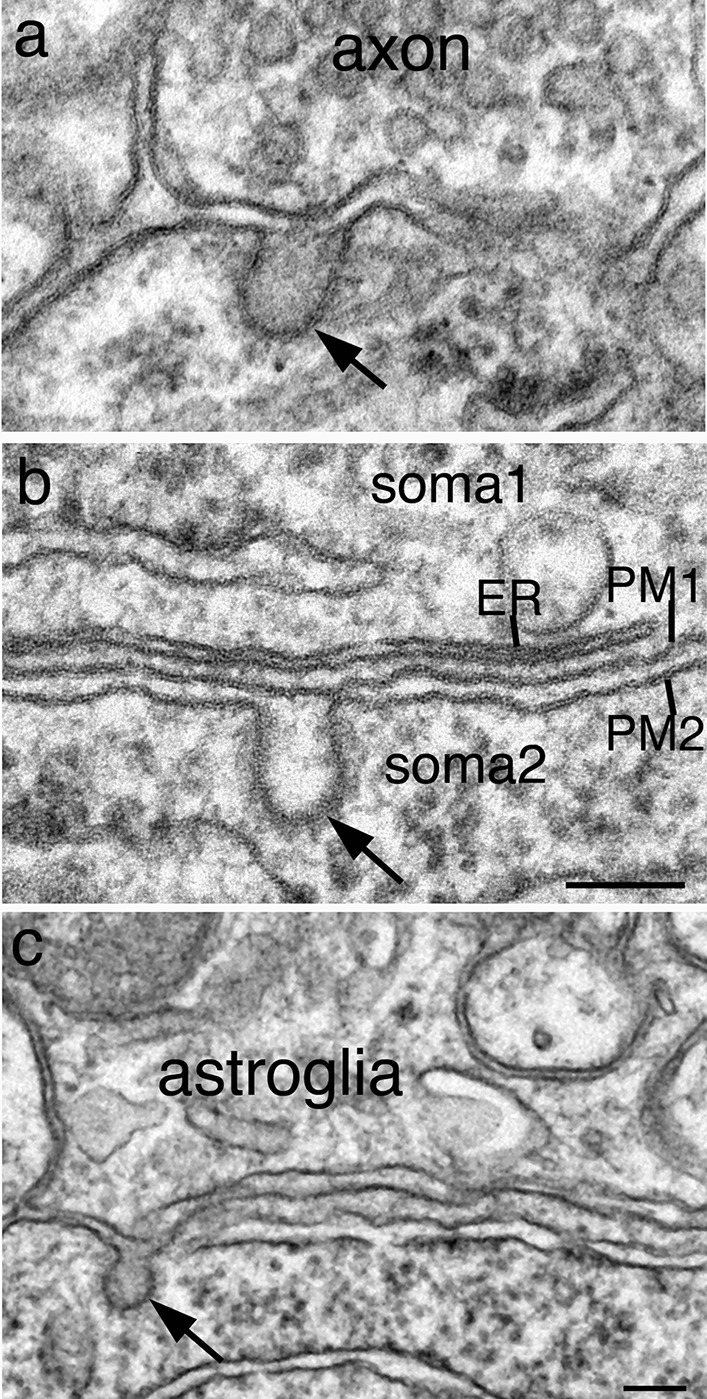
In perfusion-fixed mouse brains, somal/dendritic coated vesicles (CCV, arrows) can form juxtaposed to an axon (**a**), another soma/dendrite (**b**), or astroglia (**c**). **a**, **b** From CA1 region of the hippocampus. **b** Sampled from two neighboring pyramidal neurons where the CCV in soma2 is facing a subsurface cistern which is composed of an ER closely apposed to the plasma membrane (PM1). The CCV in **c** is sampled from a cerebellar Purkinje soma facing an astroglial process. Scale bars = 100 nm, **a**, **b** share the same scale bar

In perfusion-fixed brains, somal/dendritic CCPs were apposed to various cellular elements, including axons (Fig. 3a), another soma/dendrite (Fig. 3b), or astroglia (Fig. 3c). In dissociated cultures, somal/dendritic CCPs occurred on plasma membrane exposed to culture media without any juxtaposed cellular elements (Additional file 1b, Fig. 2a, b). Thus, formation of CCP in neuronal soma/dendrites appears to be an inherent property of the neuron, not dependent on the juxtaposed cellular elements.

### Clathrin patches on multivesicular bodies have a different structural organization than those of CCVs

**Fig. 4:**
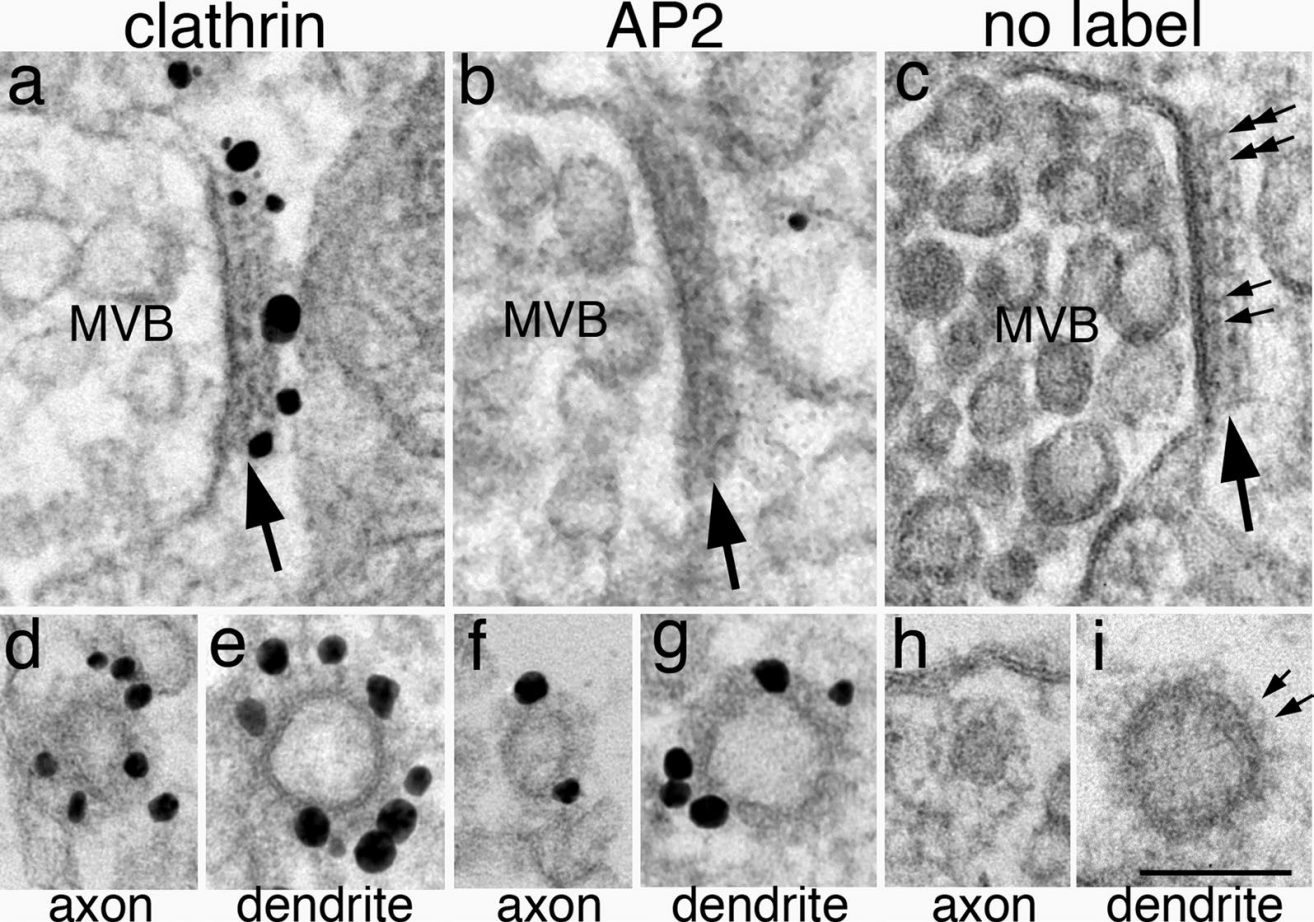
Multivesicular bodies (MVB, top row) in neurons contain a dark patch (large arrows) that label for clathrin (**a**) but not for AP2 (**b**). In contrast, in the lower row, clathrin-coated vesicles in axons (**d**, **f**) and dendrites (**e**, **g**) label for both clathrin (**d**, **e**) and AP2 (**f**, **g**). In samples fixed with glutaraldehyde for better structural preservation (no label, right column), a two layered arrangement with a uniform periodicity is visible (small arrows in **c**). The thickness of this patch is greater than those of the coated vesicles in axons (**h**) or in dendrites (**i**). MVBs in top row were sampled from neuronal soma (**a**) and dendrites (**b**, **c**). Scale bar = 100 nm

In addition to CCVs and CCPs, multivesicular body (MVB), an organelle of late endosomal origin [20], also labeled for clathrin on its limiting membrane. Label for clathrin was concentrated on a patch of dark material attached to the cytoplasmic side of the MVB (Fig. 4a, Additional file 4c). Here in dissociated hippocampal neuronal cultures, MVBs were seen throughout the neuron, like in brains [21]. It is also confirmed here that the dark patches on MVB consisted of two layers (Fig. 4c) at a thickness of ~ 30 nm [22, 23]. These patches labeled for clathrin (Fig. 4a), but not for AP2 (Fig. 4b), findings consistent with those reported in non-neuronal cells [23]. MVBs in astroglia displayed similar features as in neurons (Additional file 4). Notably, the labeling pattern of clathrin on MVB is different from those of CCVs in axons (Fig. 4d, f) or in dendrites (Fig. 4e, g) where assembled clathrin molecules are evenly distributed in a single layer around the entire vesicles, and these CCVs label for both clathrin (Fig. 4d, e) and AP2 (Fig. 4f, g).

Double-layered, clathrin-labeled patches were never seen on any other organelles except on MVB. The length of these clathrin-labeled patches on MVB in a single section was typically ~ 200 nm, and in some sections could be as long as 300–400 nm (Additional file 4C). The area of such a clathrin-labeled patch is sufficient to support the formation of a CCV. However, no budding of vesicles, either into the lumen of MVB or into the cytoplasm were observed from these patches [23]. Thus, these patches are large enough to be resolved by fluorescence LM as puncta of concentrated clathrin signals, but the present evidence suggests that these patches are not involved in budding of coated vesicles.

### Increase of CCP and CCV in presynaptic axon terminals under excitatory conditions

CCPs and CCVs are preferentially located at the periphery of presynaptic active zones of frog neuromuscular junctions [8], lamprey giant axon terminals [9], and mouse dissociated cortical cultures [3]. In all three experimental systems, CCPs and CCVs are rarely seen in resting synapses but become more frequent upon stimulation. Here, archived images of perfusion-fixed adult rat and mouse brains [15, 16] were examined to see if the number of CCV and CCP in presynaptic terminals is affected by the activity state of the synapses caused by the particular perfusion fixation conditions.

**Fig. 5:**
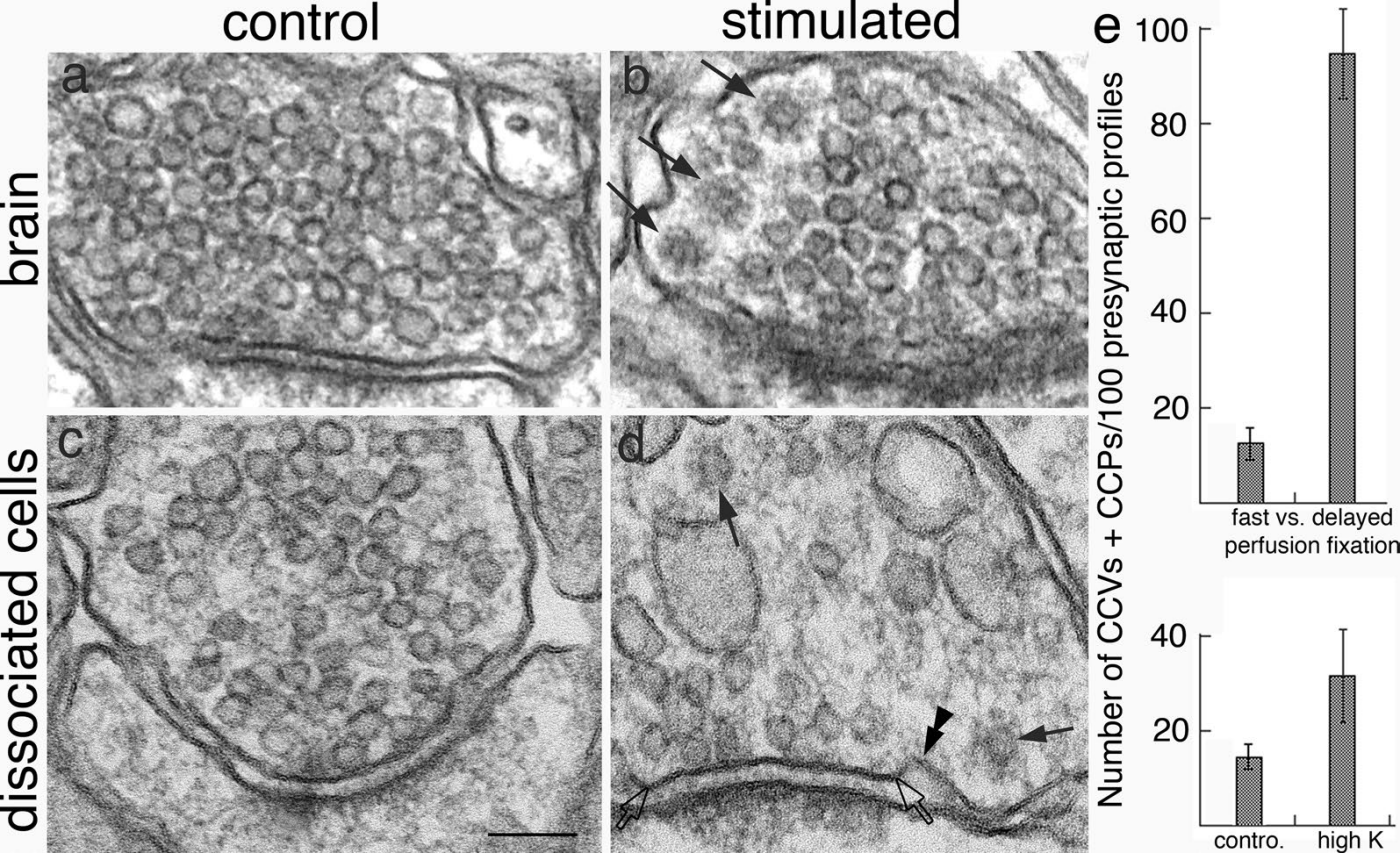
CCVs were uncommon in presynaptic terminals of fast perfusion-fixed brains (**a**), but became more abundant (arrows in **b**) upon a 5 min delay in perfusion fixation (**b**). Samples were from cerebral cortex of the mouse brain, and SVs were not noticeably depleted or dispersed in the delayed perfusion-fixed brains (**b**). Bar graphs in upper panel of **e** represent means of seven pairs of samples (data from Additional file 5A). In dissociated hippocampal cultures, upon depolarization with high K^+^, synaptic vesicles were typically dispersed and depleted (**d**). CCVs (arrows in **d**) were sometimes seen more frequently in high K^+^ (**d**) than in control samples (**c**). A CCP (double arrowheads in **d**) was seen adjacent to the active zone (between two open arrows) of the synapse. Bar graphs in lower panel of **e** represent means of four experiments (data from Additional file 5B). Scale bar = 100 nm

A 5–8 min delay in perfusion fixation has been shown to trigger activity in neurons [15, 16]. Thus, synapses are under a resting state after “fast” perfusion fixation, and under a stimulated state after “delayed” perfusion fixation, which induces ischemic stress [16]. CCVs and CCPs were consistently more abundant in delayed (Fig. 5b) than in fast perfusion-fixed brains (Fig. 5a), and these CCVs were typically located at the periphery of SV clusters (Fig. 5b). Notably, CCVs outnumber CCPs, perhaps reflecting their respective residence time. When CCVs and CCPs were scored from seven pairs of samples from different regions of the brain, their average number per 100 presynaptic profiles increased by 7.5 fold upon delayed perfusion fixation (Fig. 5e upper panel, Additional file 5A).

In 3 week-old dissociated hippocampal cultures, depolarization with high K^+^ at 90 mM for 2–3 min causes dispersion and depletion of SVs [12]. These findings are consistent with the idea that high K^+^-treated synaptic terminals are highly stimulated, resulting in massive exocytosis of SVs. However, conspicuous increase of CCVs and CCPs was only detected in some of the high K^+^-treated samples. For example, more CCVs and CCPs were observed in high K^+^-treated samples (Fig. 5d) than in controls (Fig. 5c) in some experiments (exp 1 and 2 in Additional file 5B) but not in others (exp 3 and 4 of Additional file 5B). Bar graphs in lower panel of Fig. 5e represent means of 4 experiments. Since exocytosis takes place in milliseconds and endocytosis requires minutes [18], It is possible that the formation of CCVs needs more time than the acute 2–3 min of treatment carried out in the present study. Indeed, a previous EM study of dissociated mouse neuronal cultures reported an increase of CCVs in presynaptic terminals upon 10 min of high K^+^ treatment [24].

### Depolarization induces redistribution of clathrin in presynaptic axon terminals

**Fig. 6:**
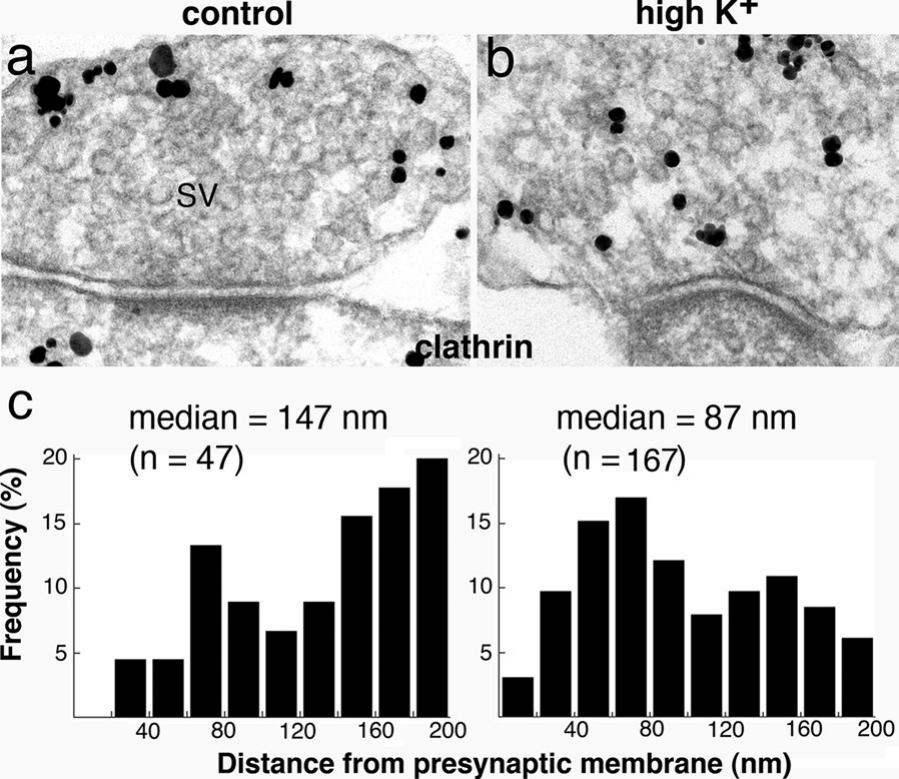
Depolarization induces redistribution of clathrin in presynaptic axon terminals of dissociated hippocampal cultures. Under control conditions, label for clathrin was concentrated outside of the SV clusters (**a**). Upon high K^+^ treatment, SVs became de-clustered, and label for clathrin became dispersed among the SVs (**b**). Distance measurements of label for clathrin showed a shift toward the presynaptic membrane upon high K^+^ treatment (**c,** representative histograms of data from exp 1 of Additional file 6)

Distribution of clathrin molecules was studied by pre-embedding immunogold labeling of 3 week-old dissociated hippocampal cultures. Under control conditions, label for clathrin was absent from the active zone and typically concentrated outside of SV clusters (Fig. 6a, Additional file 2). Upon depolarization with high K^+^, label for clathrin became dispersed among the de-clustered SVs (Fig. 6b).

Measurement of density of label for clathrin within 200 nm of the presynaptic membrane showed low labeling densities, ~ 2–5 particles per µm of active zone (Additional file 6). Upon high K^+^ treatment, the density increased by ~ 3.7 fold, on average (Additional file 6). The median distance of clathrin label, averaged from 3 experiments, decreased from 150 nm under control conditions to 88 nm upon high K^+^ treatment (Additional file 6). Figure 6c shows histograms of distance measurement from one representative experiment. These results indicate that upon depolarization, more clathrin molecules moved into the measurement area within 200 nm of the presynaptic membrane.

### Depolarization induces a decrease of CCPs and CCVs in soma/dendrites

**Fig. 7:**
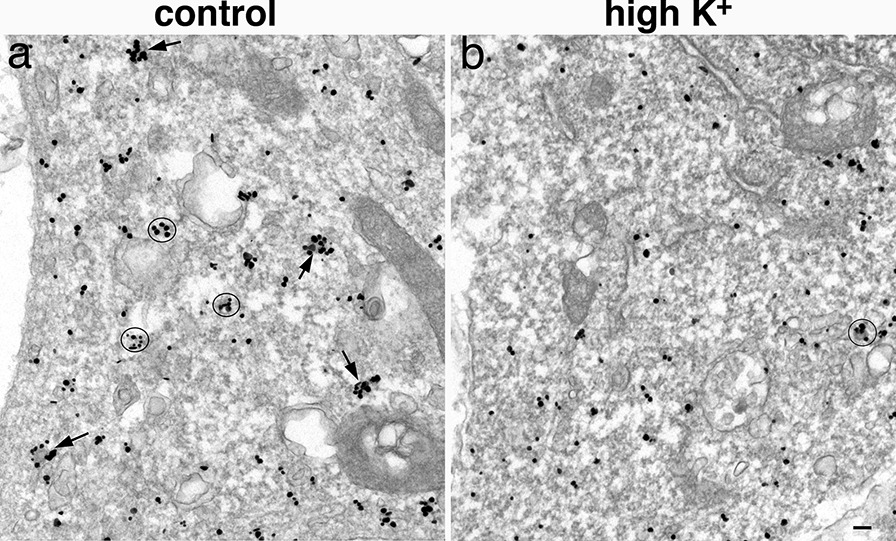
Distribution of label for clathrin in neuronal soma under control (**a**) and depolarization conditions (**b**). There were many more tightly clustered labels under control conditions (arrows and circles in **a**) than under high K^+^ treatment (**b**). Scale bar = 100 nm

In neuronal soma and dendrites under control conditions, the most striking feature of label for clathrin was the abundant clusters of aggregated labels (arrows and circles in Fig. 7a), which was lacking in axons. Serial section analysis revealed that many such clusters of tightly aggregated clathrin labels are indeed CCVs sectioned at the edge of vesicles (Additional file 3). Thus, each tightly aggregated clathrin labels can be reasonably assumed to represent a CCV. Notably, many clathrin labels also appeared as individual particles representing unassembled clathrin molecules dispersed in the cytoplasm [5, 7, 10].

Upon depolarization with high K^+^, the tightly aggregated label for clathrin disappeared, and the great majority of clathrin labels appeared as individual particles (Fig. 7b). Likewise, the number of clathrin-labeled CCPs and CCVs near plasma membrane of neuronal soma/dendrites decreased to ~ 28% of control values (Additional file 7). The disappearing of tightly aggregated clathrin labels in the cytoplasm suggests that clathrin molecules disassembled from CCVs upon depolarization.

Whether depolarization also induced a decrease in clathrin-mediated endocytosis (CME) was tested in another set of experiments where 3 week-old dissociated cultures were fixed with glutaraldehyde for better structural preservation. Plasma membrane of neuronal somas were traced to score the number of CCPs, which were identified by their characteristic coat on the omega figure (Additional file 1b), and which represent bona fide CME. The number of CCPs decreased to ~ 42% of control values upon depolarization (Additional file 8). These results indicate that in addition to increased disassembly of clathrin from CCVs, depolarization also induced a decrease in CME in neuronal soma.

### Number of peri-PSD CCP is not significantly affected by depolarization

**Fig. 8:**
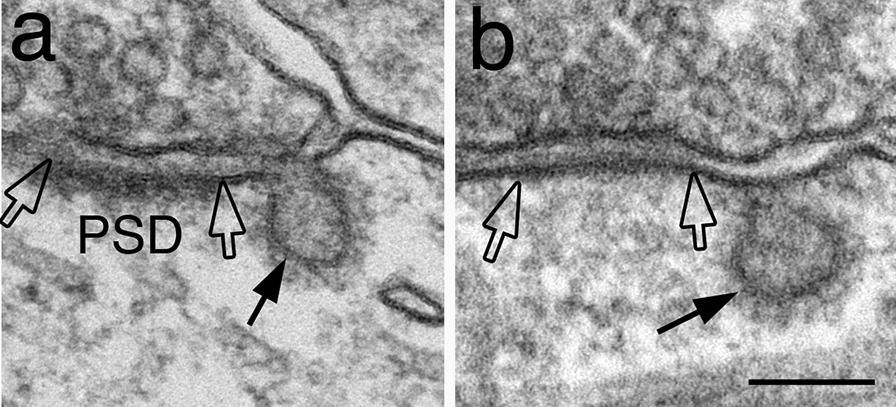
CCP at peri-PSD locations. A CCP (arrow in **a**) is located adjacent to the PSD (the edges of which are marked by open arrow) of a glutamatergic excitatory synapse. Similar pits (arrow in **b**) also exist at inhibitory synapses, where the postsynaptic membrane (area between open arrows) lacks the asymmetric density at the synaptic junction. Both synapses are sampled from perfusion-fixed mouse hippocampus. Scale bar = 100 nm

It has been proposed that there are specialized “endocytic zones” near synapses in spines that may facilitate the internalization of glutamate receptors [5, 6]. However, there are also reports that suggest CCPs near postsynaptic densities (PSD) may not be particularly involved in endocytosis of glutamate receptors [7]. In the present EM study, I defined only CCPs located immediately adjacent to (within 30 nm of) the PSD as being peri-PSD (Fig. 8a), a definition different from the “endocytic zone” reported by previous LM studies which included clathrin puncta within 300 nm of the PSD [6]. Notably, peri-PSD CCP existed in both excitatory (Fig. 8a) and inhibitory (Fig. 8b) synapses. It should also be noted that no CCP was ever detected at the PSD itself [7, 10] or the inhibitory postsynaptic specialization, indicating that clathrin cannot assemble at these specialized postsynaptic junctional membranes, and that the closest site where CME can take place is at these peri-synaptic locations.

The number of peri-PSD pits of glutamatergic excitatory synapses was scored from archived images [13] of dissociated cultures to see if depolarization induces any change in the occurrence frequency of these peri-PSD pits. The number pooled from 10 experiments did not change (Additional file 9) between control and high K^+^-treated samples (15.4 vs. 14.6 peri-PSD pits/1000 synaptic profiles, respectively).

## Discussion

The present EM study examines stimulation-induced differential changes of clathrin-coated vesicles and pits (CCV and CCP) in axons compared to soma/dendrites, and depolarization-induced redistribution of clathrin in these two polarized neuronal compartments.

CCVs are larger in soma/dendrites than in axon terminals [7]. Because axons and dendrites are polarized early in development with different cytoplasmic contents and PM compositions [19, 25], these different components may contribute to the size difference of CCVs in these two polarized compartments. Since two key proteins of the coat of CCV, clathrin and AP2, are present in both axonal and dendritic CCVs, it is unlikely that these two proteins play pivotal roles in this size difference. Whether other adaptor and accessory proteins, and/or cargos could determine the size of CCVs in axons vs. dendrites awaits future experiments with genetic manipulations.

CCVs in presynaptic terminals are mostly present near SV clusters, playing an important role in SV recycling [3, 4]. Although the initial endocytosis in axon terminals could occur through different modes such as CME, bulk endocytosis or ultrafast endocytosis, the final steps of SV formation all involve the shedding of clathrin from CCVs, which are the same size as SVs at ~ 40 nm [4]. These findings suggest a precise mechanism within presynaptic endosomes that controls the size and composition of CCVs, which will eventually shed their clathrin coat to become SVs [4, 18, 24]. Interestingly, in axons of dissociated cultures, especially the younger cells at 4 DIV, there was a distinct class of CCVs with a larger diameter at ~ 70 nm. These larger CCVs in axons seem unlikely to be involved in SV recycling, but serve other functions that are more prominent during development. I suggest that they may be involved in axon transport of clathrin [26].

In presynaptic terminals under resting conditions, clathrin molecules are dispersed in cytoplasm unassembled, excluded from SV clusters and concentrated at the periphery of SV clusters. These concentrated clathrin molecules may be poised for ready recruitment to form CCPs near PM or endosomes in presynaptic terminals [24]. Upon depolarization by high K^+^ treatment, label for clathrin became dispersed among the de-clustered SVs, as if a barrier was broken and unassembled clathrin molecules can now passively diffuse and move closer to the active zone. The functional implication for this activity-induced redistribution of clathrin in presynaptic terminals is not clear.

On the other hand, CCVs in soma/dendrites are involved in constitutive internalization of nutrients, and in regulated internalization of transmitter receptors [5, 6]. Interestingly, virtually all CCPs on somal/dendritic PM, contain ferritin [22] and TfR [13], two proteins involved in iron uptake. However, the size of dendritic CCV ranged fairly widely from 70 to 110 nm. Thus, the possibility that there are more than one population of somal/dendritic CCVs cannot be excluded, and that the size of CCV could be determined by different cargos, receptors and/or adaptors.

In soma/dendrites under resting conditions, clathrin-labeled CCVs were much more prevalent than in axon terminals. Upon depolarization, the number of CCPs and CCVs in soma/dendrites significantly decreased, most likely resulting from a decrease in CME and a heightened shedding of clathrin from CCVs. Both processes could result in augmentation of receptor concentration on the PM by reducing internalization of receptors and by facilitating the recycling of receptors back to the PM. Notably, depolarization induced an increase in concentration of the AMPA subtype of glutamate receptors on PM of neuronal soma [13]. Thus, more receptors may be available for lateral diffusion into the synaptic locations affecting synaptic signaling [27]. Indeed, depolarization also induces a reversible increase in the concentration of AMPA receptors at PSDs [13]. The decrease of synaptic receptors during the recovery period could be achieved by direct endocytosis of receptors near PSD [5, 6] and/or by lateral diffusion of receptors out of the PSD and then be endocytosed in surrounding PM away from synapses [5, 7, 10, 27].

The presence of “endocytic zones” near synapses (GFP-clathrin puncta within spines) has been proposed to represent a specialization dedicated to endocytosis near the postsynaptic membrane [5]. A subsequent EM study illustrates that three major proteins for CME, clathrin, AP2 and dynamin, are indeed present in spine heads lateral to PSDs, although CCPs immediately adjacent to PSDs are rare [10]. The present study focused on the analysis of “peri-PSD” CCP (pits that are within 30 nm of the edge of the PSD), and confirmed that their occurrence frequency is relatively low at ~ 1.5% of synaptic profiles examined in single thin sections. To date, various LM [5, 6, 28] and EM studies [13] have produced different results on whether activity or NMDA treatment induces changes in endocytosis near synapses. While different experimental conditions could account for some of the different findings, different definition of “endocytic zone” in LM studies (within 300 nm of PSD [6]) vs. “peri-PSD CCP” in EM studies may contribute as well. A larger distance between CCP and PSD would have allowed inclusion of more CCPs to be classified as near synapses. Thus, the issue of whether these CME locations near synapses are preferred in internalizing glutamate receptors under different conditions still awaits further investigation, perhaps through correlative studies combining different techniques.

## Supplementary information

**Additional file 1:** Methods on size measurement and scoring of CCV/CCP in soma/dendrites.

**Additional file 2:** Measurement for density and distance of label for clathrin at presynaptic terminals.

**Additional file 3:** Serial sections (# 1-5) of a neuronal soma labeled for clathrin.

**Additional file 4:** Multivesicular body in astrocytes (a, b) and neuron (c).

**Additional file 5:** Number of CCVs and CCPs per 100 presynaptic profiles in perfusion-fixed brains (5A) and dissociated cultures (5B).

**Additional file 6:** Density and median distance of label for clathrin at presynaptic terminals under control and depolarizing conditions.

**Additional file 7:** Number of clathrin-labeled CCVs and CCPs measured at 1 μm depth of cytoplasm from PM / μm PM under control and depolarizing conditions.

**Additional file 8:** Number of CCPs per neuronal soma under control and depolarizing conditions.

**Additional file 9:** Number of peri-PSD CCP under resting and depolarizing conditions.

## Data Availability

The datasets generated and/or analyzed during the current study are available from the corresponding author on reasonable request.

## Abbreviations

AP2: Adaptor protein-2
CCV: Clathrin-coated vesicle
CCP: Clathrin-coated pit
CME: Clathrin-mediated endocytosis
GFP: Green fluorescent protein
MVB: Multivesicular body
PM: Plasma membrane
PSD: Postsynaptic density
SEP: Super-ecliptic pHluorin
SV: Synaptic vesicles
TfR: Transferrin receptor
