# Supplementary material for: Stimulation-induced differential redistributions of clathrin and clathrin-coated vesicles in axons compared to soma/dendrites
Source: Mol Brain. 2020 Oct 16;13:141. doi: 10.1186/s13041-020-00683-5 (PMC7565815; doi:10.1186/s13041-020-00683-5)
Supplement: Supplementary file 1 — Additional file 1: Methods on size measurement and scoring of CCV/CCP in soma/dendrites. [file 13041_2020_683_MOESM1_ESM.pdf]

Additional File 1. Methods on size measurement and scoring of CCV/CCP in soma/dendrites.

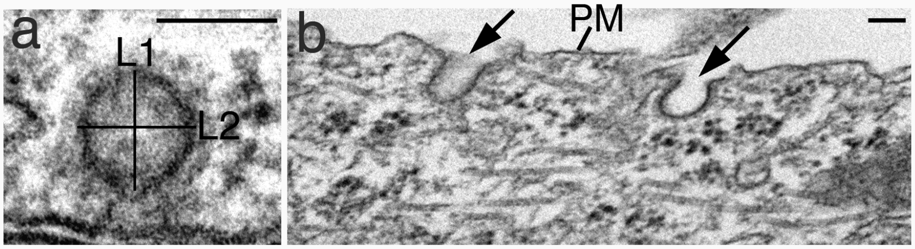

(a) Average diameter of CCV = (L1 + L2) / 2.

(b) Clathrin-coated pits (CCP) on plasma membrane (PM) of neuronal somas were identified by the characteristic coats on the cytoplasmic side of the omega figures (arrows). Scale bars = 100 nm.
