# Supplementary material for: Stimulation-induced differential redistributions of clathrin and clathrin-coated vesicles in axons compared to soma/dendrites
Source: Mol Brain. 2020 Oct 16;13:141. doi: 10.1186/s13041-020-00683-5 (PMC7565815; doi:10.1186/s13041-020-00683-5)
Supplement: Supplementary file 2 — Additional file 2: Measurement for density and distance of label for clathrin at presynaptic terminals. [file 13041_2020_683_MOESM2_ESM.pdf]

Additional File 2. Measurement for density and distance of label for clathrin at presynaptic terminals.

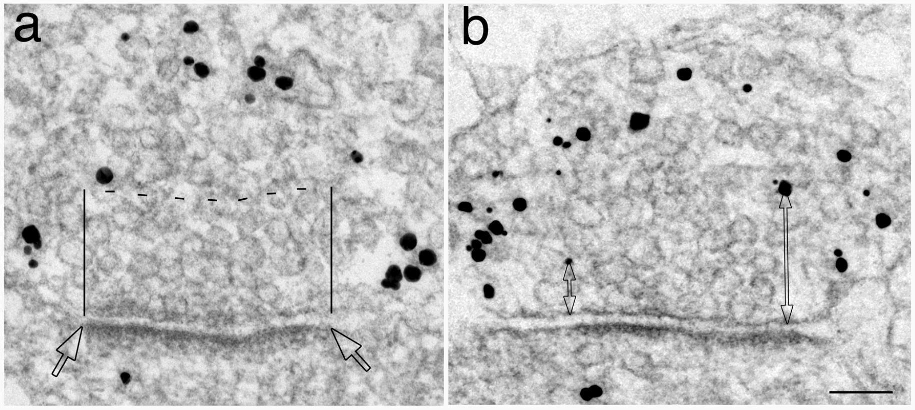

Samples were 3 wk-old dissociated hippocampal cultures fixed under control conditions and labeled with a clathrin antibody.

Only cross-sectioned synaptic profiles were included for measurement.

Boxed area in (a) includes presynaptic area that is 200 nm deep from the presynaptic membrane that faces the PSD (edges were marked by two open arrows). Black particles of heterogeneous size are silver enhanced gold particles that represent label for clathrin. All black particles within this measurement area were counted for density and distance (length of the open arrows in b) of label. Scale bar = 100 nm.
