# Supplementary material for: Stimulation-induced differential redistributions of clathrin and clathrin-coated vesicles in axons compared to soma/dendrites
Source: Mol Brain. 2020 Oct 16;13:141. doi: 10.1186/s13041-020-00683-5 (PMC7565815; doi:10.1186/s13041-020-00683-5)
Supplement: Supplementary file 3 — Additional file 3: Serial sections (# 1-5) of a neuronal soma labeled for clathrin. [file 13041_2020_683_MOESM3_ESM.pdf]

Additional File 3. Serial sections (# 1-5) of a neuronal soma labeled for clathrin.

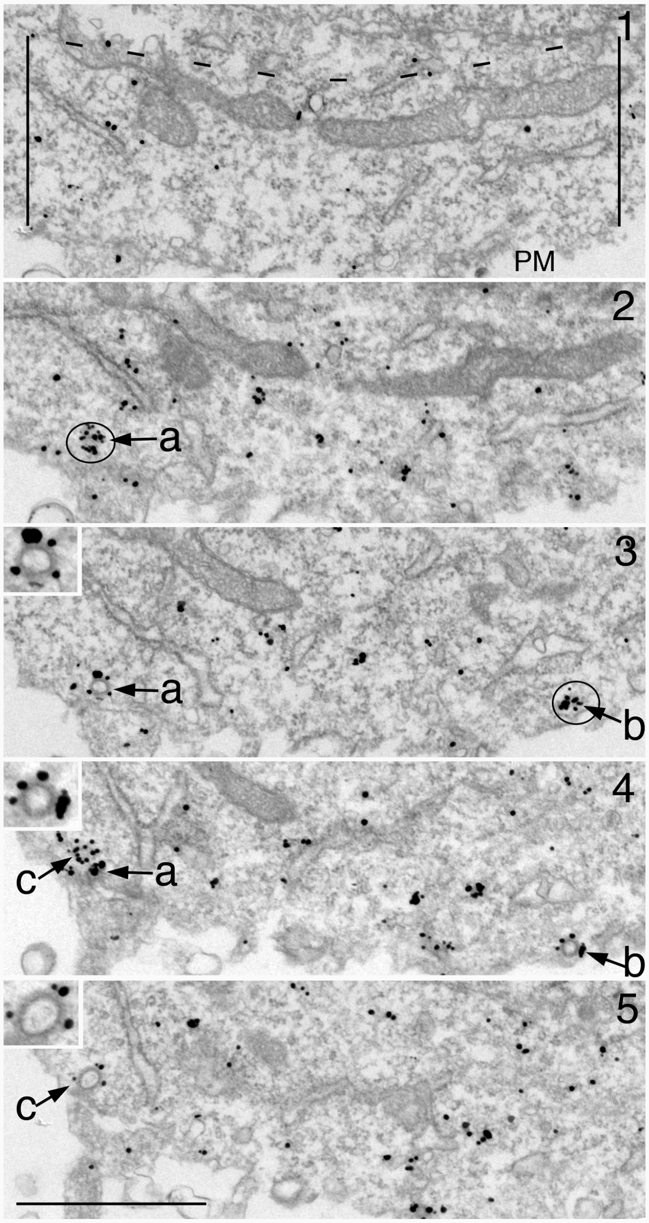

For measurement of number of CCVs and CCPs near plasma membrane (PM), a band of cytoplasm within 1 μm from the PM was included for measurement (area marked by PM and the dashed line in section #1). CCVs (marked as “a” in section #3, “b” in section #4, and “c” in section #5; enlarged as insets) existed in 2-3 serial sections. The adjacent sections to these clear-cut CCVs typically contained tightly clustered labels for clathrin (circled in sections # 2 and #3, with more than 5 particles within a 100 nm area). Scale bar = 1 μm.
