# Supplementary material for: Stimulation-induced differential redistributions of clathrin and clathrin-coated vesicles in axons compared to soma/dendrites
Source: Mol Brain. 2020 Oct 16;13:141. doi: 10.1186/s13041-020-00683-5 (PMC7565815; doi:10.1186/s13041-020-00683-5)
Supplement: Supplementary file 4 — Additional file 4: Multivesicular body in astrocytes (a, b) and neuron (c). [file 13041_2020_683_MOESM4_ESM.pdf]

Additional file 4. Multivesicular body in astrocytes (a, b) and neuron (c).

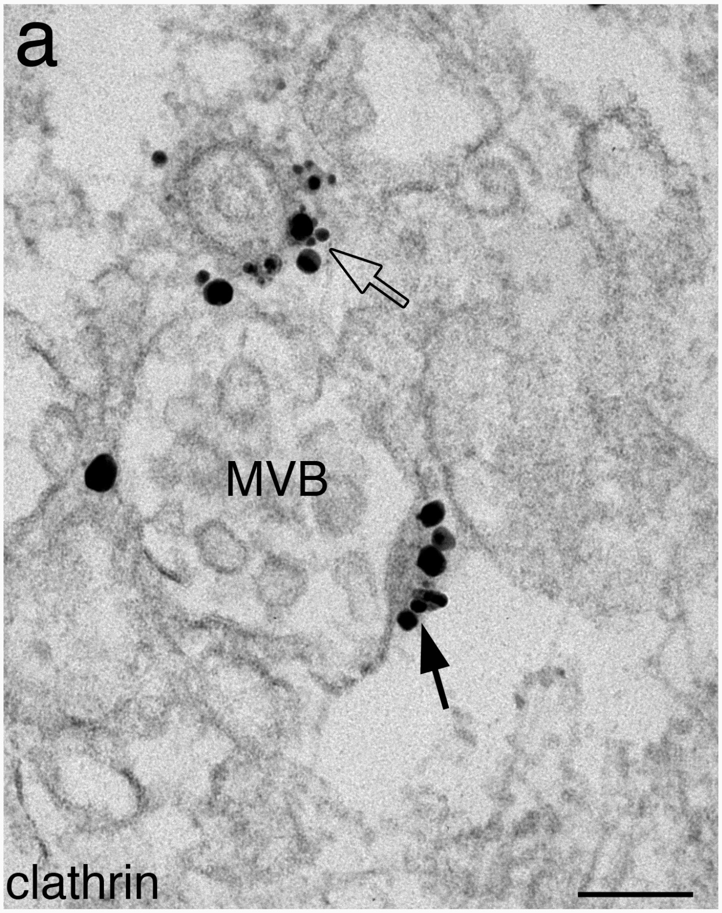

(a) A multivesicular body (MVB) with a patch of clathrin labeling (black arrow) on its limiting membrane. Open arrow points to a nearby clathri-coated vesicle. Image from cytoplasm of an astrocyte in dissociated hippocampal cultures.

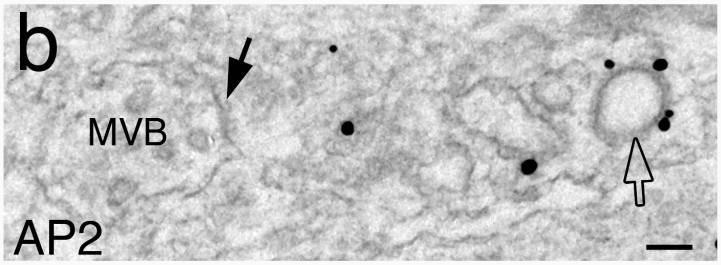

(b) The clathrin patch of MVB in astrocyte does not label for AP2 (black arrow). However, clathrin-coated vesicle does label for AP2 (open arrow). Scale bars = 100 nm.

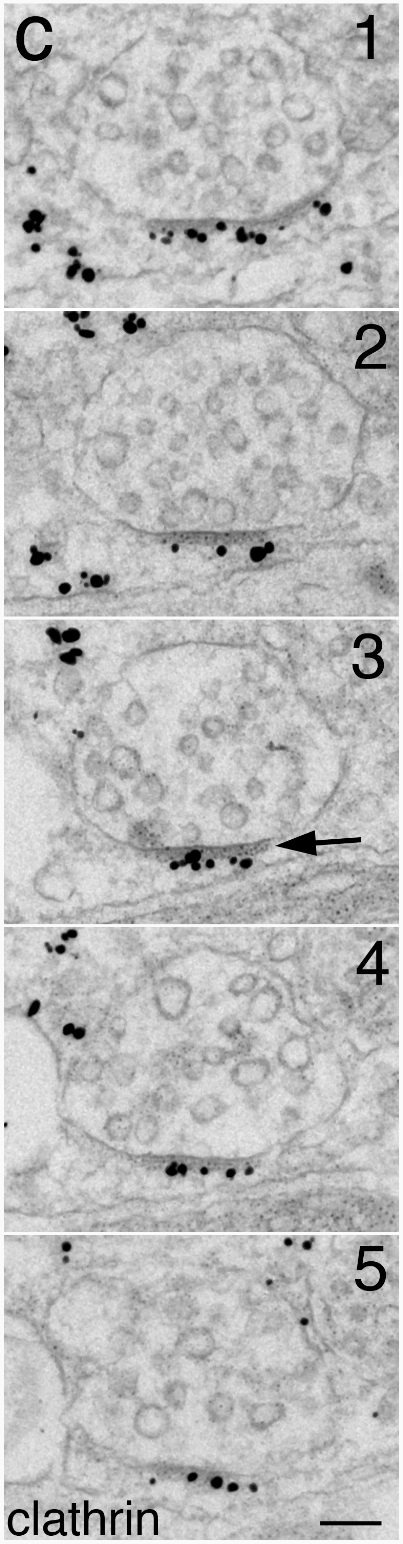

(c) Serial sections (1-5) of a clathrin-labeled MVB in a neuronal dendrite.
