# Supplementary material for: Stimulation-induced differential redistributions of clathrin and clathrin-coated vesicles in axons compared to soma/dendrites
Source: Mol Brain. 2020 Oct 16;13:141. doi: 10.1186/s13041-020-00683-5 (PMC7565815; doi:10.1186/s13041-020-00683-5)
Supplement: Supplementary file 5 — Additional file 5: Number of CCVs and CCPs per 100 presynaptic profiles in perfusion-fixed brains (5A) and dissociated cultures (5B). [file 13041_2020_683_MOESM5_ESM.pdf]

Additional File 5. Number of CCVs and CCPs per 100 presynaptic profiles in perfusion-fixed brains (5A) and dissociated cultures (5B).

**5A**	**Fast perfusion**	**Delayed perfusion**
Rat cerebral cortex	4.7 (43)	79.2 (48)
Mouse cerebral cortex	5.9 (51)	98.9 (47)
	28.6 (35)	135.3 (34)
Mouse cerebellum	6.8 (44)	61.1 (54)
	21.1 (38)	116.7 (18)
Mouse hippocampus CA1	12.7 (55)	95.7 (46)
	7.5 (67)	76.9 (26)
**Mean ± SEM**	**12.5 ± 3.4**	**94.8 ± 9.6**<br>**P< 0.0001, paired t**

**5B**	**control**	**High K^+**
Exp 1	10 (40)	49.1 (53)
Exp 2	10 (50)	48.1 (54)
Exp 3	16.7 (30)	14.3 (21)
Exp 4	21.2 (33)	15.2 (33)
**Mean ± SEM**	**14.5 ± 2.7**	**31.7 ± 9.8**<br>**NS, paired t**

(n) = number of presynaptic profiles scored.

NS, not significant.
