# Supplementary material for: Stimulation-induced differential redistributions of clathrin and clathrin-coated vesicles in axons compared to soma/dendrites
Source: Mol Brain. 2020 Oct 16;13:141. doi: 10.1186/s13041-020-00683-5 (PMC7565815; doi:10.1186/s13041-020-00683-5)
Supplement: Supplementary file 6 — Additional file 6: Density and median distance of label for clathrin at presynaptic terminals under control and depolarizing conditions. [file 13041_2020_683_MOESM6_ESM.pdf]

Additional File 6. Density and median distance of label for clathrin at presynaptic terminals under control and depolarizing conditions.

	**Density** [number of labels within 200 nm of PM /µm presynaptic membrane]			**Median Distance** [nm, from presynaptic membrane]	
	**control**	**High K^+**	**% control**	**control**	**High K^+**
Exp 1	3.9 ± 1.0 (n=29)	15.9 ± 2.3 (n=33)	408% P<0.0001	147 (n=47)	87 (n=147) P<0.0001
Exp 2	1.8 ± 0.6 (n=20)	7.9 ± 1.0 (n=25)	439% P<0.0001	177 (n=12)	87 (n=84) P<0.005
Exp 3	4.7 ±1.2 (n=25)	11.8 ± 3.1 (n=16)	251% P<0.05	127 (n=35)	90 (n=56) Not significant
**Mean ± SEM (Paired t)**			**366 ± 58% P<0.05**	**150 ± 15**	**88 ± 1 P<0.1**

For density of label, (n)= number of synaptic profiles measured; for distance of label, (n) = number of labels measured.

Statistical analysis within each experiment: Student's t-test for density; Wilcoxon test for median distance.
