# Supplementary material for: Stimulation-induced differential redistributions of clathrin and clathrin-coated vesicles in axons compared to soma/dendrites
Source: Mol Brain. 2020 Oct 16;13:141. doi: 10.1186/s13041-020-00683-5 (PMC7565815; doi:10.1186/s13041-020-00683-5)
Supplement: Supplementary file 7 — Additional file 7: Number of clathrin-labeled CCVs and CCPs measured at 1 μm depth of cytoplasm from PM / μm PM under control and depolarizing conditions. [file 13041_2020_683_MOESM7_ESM.pdf]

Additional File 7. Number of clathrin-labeled CCVs and CCPs measured at 1 µm depth of cytoplasm from PM / µm PM under control and depolarizing conditions.

	**Control**	**High K^+**
Exp 1	0.48 ± 0.06 (14)	0.14 ± 0.04 (17) P<0.0005
Exp 2	0.58 ± 0.06 (14)	0.15 ± 0.03 (18) P<0.0001
Exp 3	0.57 ± 0.06 (14)	0.15 ± 0.04 (15) P<0.0001
**Mean ± SEM**	**0.54 ± 0.03**	**0.15 ± 0.01 P<0.005 (paired t)**

(n) = number of somas sampled.

Means within each experiment tested by Student's t-test.

Methods of measurement are detailed in Additional File 3.
