# Supplementary material for: Stimulation-induced differential redistributions of clathrin and clathrin-coated vesicles in axons compared to soma/dendrites
Source: Mol Brain. 2020 Oct 16;13:141. doi: 10.1186/s13041-020-00683-5 (PMC7565815; doi:10.1186/s13041-020-00683-5)
Supplement: Supplementary file 8 — Additional file 8: Number of CCPs per neuronal soma under control and depolarizing conditions. [file 13041_2020_683_MOESM8_ESM.pdf]

Additional File 8. Number of CCPs per neuronal soma under control and depolarizing conditions

	**Control**	**High K^+**
Exp 1	6.0 ± 1.2 (10)	2.7 ± 0.9 (10)<br>$P<0.05$
Exp 2	5.3 ± 0.9 (12)	2.1 ± 0.7 (15)<br>$P<0.01$
**Mean ± SEM**	**5.7 ± 0.4**	**2.4 ± 0.3**<br>**$P<0.01$, paired t**

(n) = number of neuronal somas scored.

Means within each experiment tested by Student's t-test.
