# Supplementary material for: Stimulation-induced differential redistributions of clathrin and clathrin-coated vesicles in axons compared to soma/dendrites
Source: Mol Brain. 2020 Oct 16;13:141. doi: 10.1186/s13041-020-00683-5 (PMC7565815; doi:10.1186/s13041-020-00683-5)
Supplement: Supplementary file 9 — Additional file 9: Number of peri-PSD CCP under resting and depolarizing conditions. [file 13041_2020_683_MOESM9_ESM.pdf]

**Additional File 9. Number of peri-PSD CCP under resting and depolarizing conditions**

| | **control** | **High K$^+$** |
|---|---|---|
| Exp 1 | 3 (73) | 1 (76) |
| Exp 2 | 0 (51) | 0 (29) |
| Exp 3 | 1 (44) | 0 (40) |
| Exp 4 | 0 (53) | 0 (41) |
| Exp 5 | 1 (98) | 2 (55) |
| Exp 6 | 0 (52) | 2 (84) |
| Exp 7 | 1 (65) | 1 (58) |
| Exp 8 | 0 (102) | 0 (31) |
| Exp 9 | 2 ( 28) | 0 (38) |
| Exp 10 | 1 (17) | 1 (28) |
| **Pooled total** | 9 (583) | 7 (480) |
| **Normalized to Per 1000 synaptic profiles** | **15.4 / (1000)** | **14.6 / (1000)** |

Peri-PSD CCP is defined as clathrin-coated pits within 30 nm of the postsynaptic density.
(n) = number of synaptic profiles examined.
